# Calcific Tendinitis of the Supraspinatus Tendon in an Infant

**DOI:** 10.1155/2020/9842489

**Published:** 2020-07-02

**Authors:** Masanori Wako, Jiro Ichikawa, Kensuke Koyama, Yoshihiro Takayama, Hirotaka Haro

**Affiliations:** Department of Orthopaedic Surgery, Faculty of Medicine, University of Yamanashi, 1110 Shimokato, Chuo-shi, Yamanashi 409-3898, Japan

## Abstract

Calcific tendinitis of the supraspinatus tendon in adults is common, but it is extremely rare in children. This report presents an unusual case of a 2-year-old boy with calcific tendinitis of the supraspinatus tendon. A mother brought her 2-year-old son to our hospital with a fever and severe left shoulder pain. Examination revealed a temperature of 38.6°C accompanied by a swollen shoulder with extreme pain and restricted movement. The radiographs of his left shoulder showed a large radio-opacity in the subacrominal region, and magnetic resonance imaging showed an elongated T1 and T2 hypointense signal above the supraspinatus tendon. Although these images were suggestive of calcific tendinitis of the supraspinatus tendon, we performed an open biopsy and resection in order to differentiate between a suspected diagnosis of calcific tendinitis, which is incredibly rare within pediatric patients, and infection or a soft tissue tumor. Finally, calcific tendinitis of the supraspinatus tendon was diagnosed by pathologic experiment and successfully treated, with complete resolution of pain and movement. Because only four other pediatric cases of calcific tendinitis of the supraspinatus tendon have ever been reported, there is a lack of information on the diagnostic process, management, and treatment of such a condition in young patients. Calcific tendinitis of the supraspinatus tendon still should be considered when encountering cases with typical findings even if the patient is a child.

## 1. Introduction

Calcific tendinitis of the supraspinatus tendon is the reactive calcification of the rotator cuff tendons and one of the main causes of acute shoulder pain in adults. It is commonly found in people aged 30–60 years and its incidence is reported at 2.7%–7.5% [1–3]. It is rarely found in children; indeed, only four case reports exist on pediatric calcific supraspinatus tendinitis—in children aged 3–13 years [4–7]. Here, we report a case of a 2-year-old boy with calcific supraspinatus tendinitis, who is the youngest case reported so far in the literature.

## 2. Case Presentation

The mother of a previously healthy 2-year-old boy noticed that he did not want to move his left shoulder, so she brought him to an emergency medical center. He had no history of injury or of previous symptoms in the affected arm. At the time, they were instructed to rest at home. However, 2 days later, he developed a fever of 38°C, and the mother took him to another hospital, from where the boy was transferred to our hospital for diagnosis and treatment.

Physical examination revealed a body temperature of 38.6°C, slight swelling in his left shoulder, but the local heat of his left shoulder was almost normal. There was marked tenderness, with palpation resulting in excruciating pain over the lateral part of his left shoulder. Both passive and active movements of his left shoulder were almost completely restricted due to the severe pain on any attempt at moving it.

**Figure 1:**
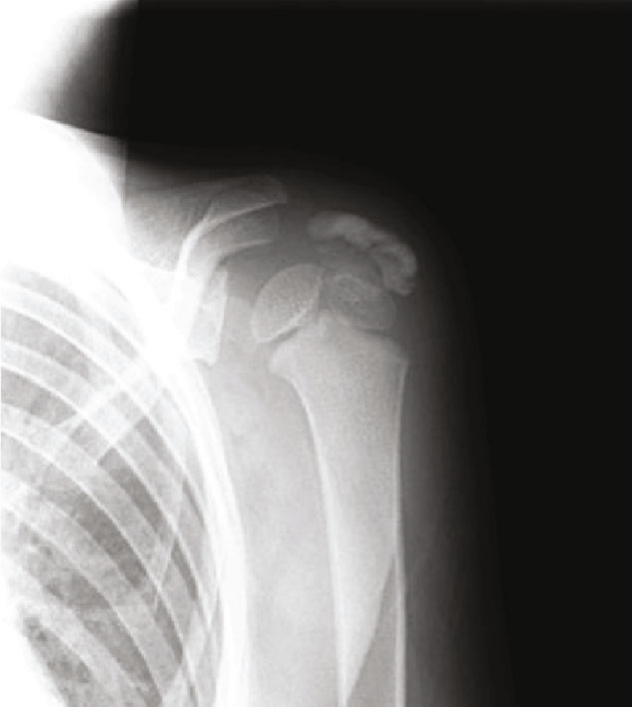
Initial radiograph of the left shoulder.

**Figure 2:**
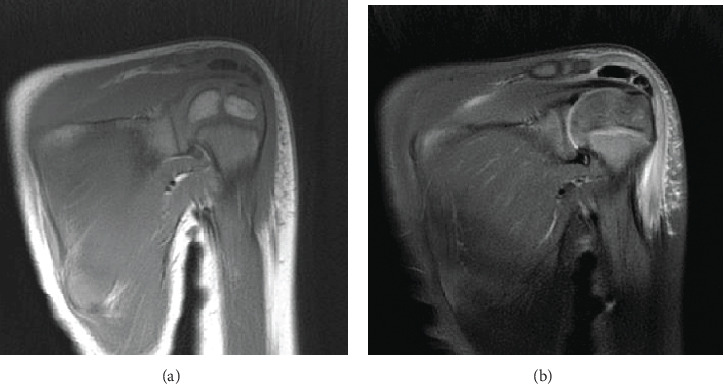
Coronal magnetic resonance imaging of the left shoulder. (a) T1-weighted; (b) T2-weighted fat suppression.

The biochemistry results revealed slightly elevated white blood cell (WBC) count at 10.77 × 10^9^/L and C-reactive protein (CRP) of 3.27 mg/dL. Radiograph of the left shoulder revealed a large ovoid radiopaque area measuring 2 × 1 cm in the subacromial region, impinged between the humerus and acromion (Figure 1). There was no obvious intralesional osseous trabeculation or cortical rim to suggest a bony origin. Noncontrast magnetic resonance imaging (MRI) of his left shoulder performed under anesthesia confirmed the ovoid lesion with a hypointense signal on both T1- and T2-weighted sequences above the supraspinatus tendon, just proximal to its insertion at the greater tuberosity (Figure 2). The imaging findings strongly suggested calcific supraspinatus tendinitis. However, to exclude infection or soft tissue tumor and establish a definitive diagnosis, we performed an open biopsy and resection.

**Figure 3:**
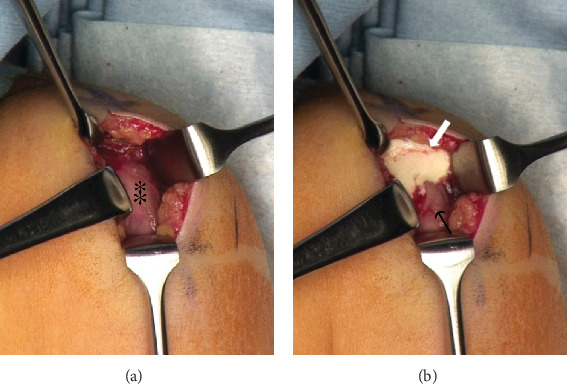
Intraoperative findings. (a) A white mass (∗) existed on the supraspinatus muscle. (b) When the capsule was incised, white paste flowed out (arrow).

Under general anesthesia, a 2-cm skin incision was made lateral to the acromion. After dividing the deltoid muscle, a capsulated white mass was noted on the supraspinatus muscle (Figure 3(a)). When the capsule was incised, white paste exuded (Figure 3(b)), which nearly confirmed the diagnosis of calcific tendinitis. We submitted the tissue for rapid intraoperative pathological examination, which showed only calcification without tumor cells or bacterium, thus confirming the diagnosis. Following this, the white capsule was removed as carefully as possible to avoid damage to the supraspinatus muscle, and after a thorough wash, we restored the deltoid muscle and closed the wound.

**Figure 4:**
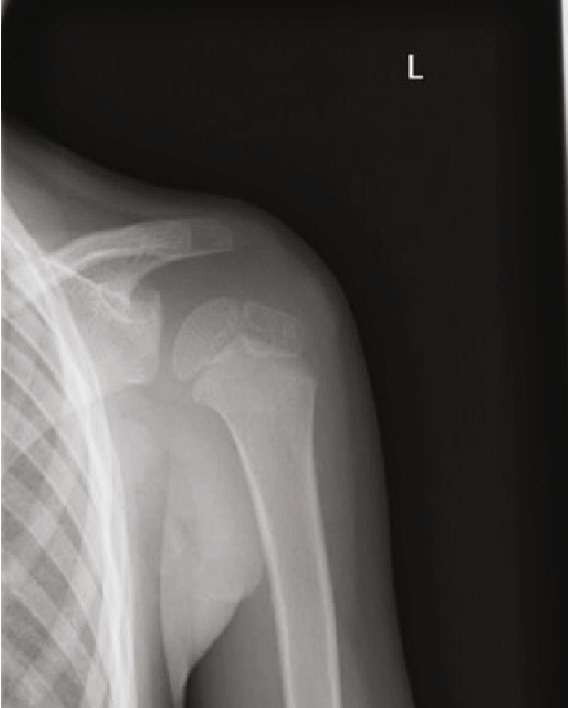
Radiograph at final follow-up.

Two days after the surgery, the patient's body temperature decreased and he could move his left shoulder without pain, so he was discharged 1 day later. Radiographs taken 2 weeks after the surgery showed that the mass had disappeared (Figure 4); the culture test was also negative. The left shoulder pain had completely subsided, and the range of shoulder motion returned to normal without any pain during movement. One year after surgery, there was no recurrence or pain and normal range of motion of the shoulder was maintained, but the operation scar was noticeable.

## 3. Discussion

We described the case of a 2-year-old boy with calcific supraspinatus tendinitis who underwent open surgical treatment with excellent postoperative course. To the best of our knowledge, this is the youngest case of calcific supraspinatus tendinitis reported so far.

Calcific tendinitis of the rotator cuff is one of the most common causes of nontraumatic pain in the shoulder. It is caused by macroscopic deposition of hydroxyapatite, a crystalline form of calcium phosphate, in the rotator cuff tendons [8]. Radiographs of the involved shoulder usually show typical calcific deposit. In general, treatment options consist of conservative therapy, such as nonsteroidal anti-inflammatory drugs, and injection of a local anesthetic with or without steroid infiltration. If conservative treatment fails, fluoroscopy-guided needle aspiration or arthroscopic surgery is typically considered. In the previous reports of pediatric calcific tendinitis of the rotator cuff, a 7-year-old boy [7] was treated conservatively, a 7-year-old girl received arthroscopic treatment [6], and a 13-year-old girl and 3-year-old boy were managed conservatively with nonsteroidal anti-inflammatory drugs [4, 5]. These reports imply that conservative treatment other may be sufficient for pediatric calcific tendinitis of the rotator cuff.

Because our patient was very young, it was difficult to determine the symptoms and the progress of the disease. Therefore, although calcific tendinitis of the rotator cuff was strongly suspected, we performed open surgery. Because we had no prior experience with a very young child with calcific tendinitis of the rotator cuff, we were not confident in our diagnosis and wanted to completely rule out pyogenic arthritis or neoplastic lesion; this is why we selected open surgery. Furthermore, 1 year after the surgery, the boy did not have shoulder pain or restriction of shoulder movement, but the operation scar was noticeable. In general, the shoulder tends to be more susceptible to scarring in children, so open surgery should be avoided in younger children as much as possible. Thus, needle aspiration or arthroscopic surgery is more desirable for pediatric calcific tendinitis of the rotator cuff than open surgery provided that the diagnosis is definitively established.

This case highlights that clinicians should be aware that calcific tendinitis of the rotator cuff can develop in pediatric patients in order to help them establish a definitive diagnosis and administer appropriate treatment.
